# Supplementary material for: NMR Spectroscopy Applied to the Metabolic Analysis of Natural Extracts of Cannabis sativa
Source: Molecules. 2022 May 30;27(11):3509. doi: 10.3390/molecules27113509 (PMC9182145; doi:10.3390/molecules27113509)
Supplement: Supplementary file 1 [file molecules-27-03509-s001.zip › molecules-1716494-supplementary.pdf]

molecules

Article

NMR Spectroscopy Applied to the Metabolic Analysis of Natural Extracts of *Cannabis sativa*

Maria Francesca Colella [1], Rosachiara Antonia Salvino [1], Martina Gaglianò [1], Federica Litrenta [2], Cesare Oliviero Rossi [1], Adolfo Le Pera [3] and Giuseppina De Luca [1,*]

[1] Department of Chemistry and Chemical Technologies (CTC), University of Calabria—UNICAL, Via P. Bucci 14C, 87036 Arcavacata di Rende, Italy; mariafrancesca.colella@unical.it (M.F.C.); rosachiara.salvino@unical.it (R.A.S.); martina.gagliano@unical.it (M.G.); cesare.oliviero@unical.it (C.O.R.)
[2] Department of Biomedical, Dental and Morphological and Functional Imaging Sciences (Biomorf), University of Messina, Polo Universitario dell'Annunziata, 98168 Messina, Italy; federica.litrenta@unime.it
[3] Calabra Maceri e Servizi s.p.a., via M. Polo 54, 87036 Rende, Italy; laboratorio@calabramaceri.it
* Correspondence: giuseppina.deluca@unical.it

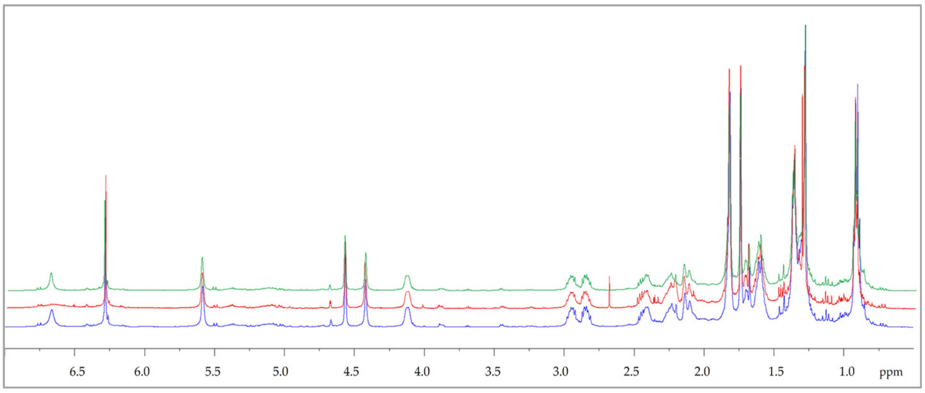

Figure S1. Comparison between ^{1}H NMR spectra of ethanol (blue), acetone (red) and hexane (green) extracts for *Tiborszallasi* variety.

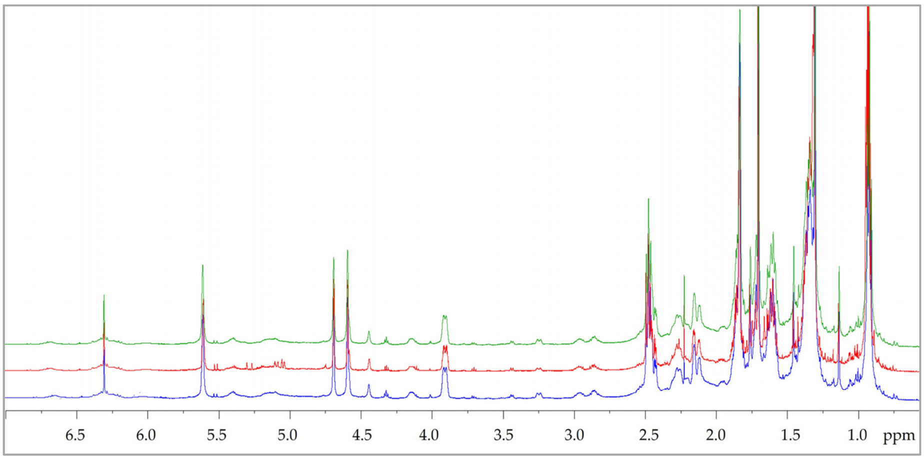

Figure S2. Comparison between ^{1}H NMR spectra of ethanol (blue), hexane (red) and acetone (green) extracts for *Kompolti* variety.

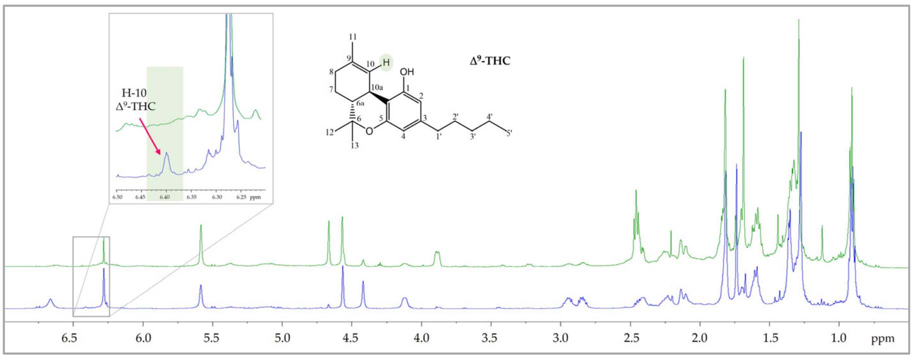

Figure S3. Comparison between the enlarged region [6.25 ppm - 6.5ppm] of the ^{1}H NMR spectra from hexane extract of *Tiborszallasi* (blue) and *Kompolti* (purple) variety. A broad peak isolated at 6.40 ppm corresponding to the proton H-10 of Δ^{9}-THC appears in the proton spectra of *Tiborszallasi* while this signal was undetectable in the ^{1}H NMR spectrum acquired for *Kompolti*.

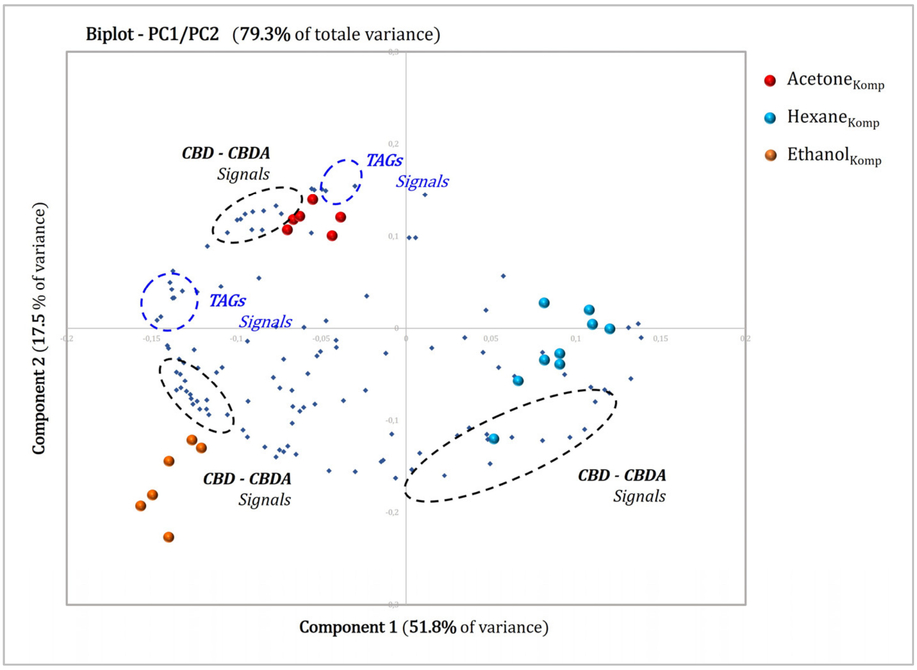

Figure S4. Biplot of PCA carried out on NMR spectra of acetone (red dots), hexane (blue dots) and ethanol (orange dots) extracts of *Kompolti* variety of hemp. The scores plot showing the first two PCs (PC1 and PC2) with their respective variation. R2X(PC1) = 51.8%, R2X(PC2) = 17.5%. .

Table S1. The ^{1}H NMR data of main cannabinoids in *Kompolti* inflorescences.

Compound	qNMR on Flowers UAE Extracts			
	qNMR IS	Hexane	Acetone	Ethanol
**CBDA** content*	Anthracene	0.4 ± 0.3	0.4 ± 0.2	0.5 ± 0.2
	Benzoic acid	0.6 ±0.2	0.5 ± 0.1	0.4± 0.1
	TMSP-d4	0.4 ± 0.3	0.4 ± 0.3	0.5 ± 0.3
**CBD** content*	Anthracene	5.7 ±0.2	5.4 ± 0.3	5.2 ± 0.2
	Benzoic acid	5.9±0.2	5.2 ± 0.2	5.3 ± 0.1
	TMSP-d4	5.9±0.2	5.6 ± 0.3	5.3 ± 0.3
**Δ^{9}-THC** content*	Anthracene<br>Benzoic acid<br>TMSP-d4	<LOD	<LOD	< LOD

*% on dry weight.
